# Home- and Community-Based Services Use Patterns and Functional Improvement among Older Care Recipients in Taiwan

**DOI:** 10.1093/geroni/igab046.3267

**Published:** 2021-12-17

**Authors:** Hsiao-Wei Yu, Shih-Cyuan Wu, Ya-Mei Chen

**Affiliations:** 1 Chang Gung University of Science and Technology, Taoyuan City, Taoyuan, Taiwan (Republic of China); 2 National Taiwan University, Taipei, Taipei, Taiwan (Republic of China)

## Abstract

The new version of Taiwan’s 10-Year Long-Term Care Plan launched in 2016 aims to reinforce the integration of home- and community-based services (HCBS). The underlying HCBS use patterns and effectiveness of functional improvement among care recipients merit investigation. The purpose of the study was to examine the association of HCBS and changes in ADLs among care recipients with different levels of disabilities in Taiwan. We accessed the sub data of Taiwan’s Long-Term Care Services Management Online System. Samples were aged 65 and over and had completed records of baseline and reassessment information during 2018 (N = 4787). Latent class analysis and multivariate linear regression were applied to examine the relationship of HCBS and functional changes. Four HCBS subpatterns were found: home-based personal care services (home-based PS) (59.16%), home-based reablement services (home-based RS) (23.90%), home-based multiple services (home-based MS) (11.93 %), and community-based services (5.01%). In the cases with mild disabilities at baseline, recipients receiving home-based RS had higher probabilities of improving in ADLs among four HCBS subgroups (for example: β = 2.65, SE = 1.19 in comparison to home-based PS). Care recipients with moderate-to-severe disability at baseline, ADLs improvement was only found in home-based PS (β = 1.63, SE = 0.82 in comparison of home-based MS). In the cases with profound disabilities, home-based PS showed positive effects on ADLs improvement (β = 2.45, SE = 0.80 in ADLs, compared to home-based RS). The study suggested that HCBS subpatterns had different impacts on older adults with different disability levels.

